# Construction of gastric cancer patient-derived organoids and their utilization in a comparative study of clinically used paclitaxel nanoformulations

**DOI:** 10.1186/s12951-022-01431-8

**Published:** 2022-05-18

**Authors:** Jiale Zou, Shuang Wang, Ningli Chai, Hua Yue, Peng Ye, Peilin Guo, Feng Li, Bo Wei, Guanghui Ma, Wei Wei, Enqiang Linghu

**Affiliations:** 1 Department of Gastroenterology and Hepatology, The First Medical Centre, Chinese PLA General Hospital, Beijing, 100853 People’s Republic of China; 2 State Key Laboratory of Biochemical Engineering, Institute of Process Engineering, Chinese Academy of Sciences, Beijing, 100190 People’s Republic of China; 3 School of Chemical Engineering, University of Chinese Academy of Sciences, Beijing, 100049 People’s Republic of China; 4 Department of General Surgery, The First Medical Centre, Chinese PLA General Hospital, Beijing, 100853 People’s Republic of China

**Keywords:** Gastric cancer, Patient-derived organoids, Nanoformulation, Albumin-bound paclitaxel, Liposomal paclitaxel

## Abstract

**Background:**

Gastric cancer (GC) is a highly heterogeneous disease with many different histological and molecular subtypes. Due to their reduced systemic adverse effects, nanoformulation agents have attracted increasing attention for use in the treatment of GC patients in the clinic. To improve therapeutic outcomes, it is vitally necessary to provide individual medication references and guidance for use of these nanoformulations, and patient-derived organoids (PDOs) are promising models through which to achieve this goal.

**Results:**

Using an improved enzymatic digestion process, we succeeded in constructing GC PDOs from surgically resected tumor tissues and endoscopic biopsies from GC patients; these PDOs closely recapitulated the histopathological and genomic features of the corresponding primary tumors. Next, we chose two representative paclitaxel (PTX) nanoformulations for comparative study and found that liposomal PTX outperformed albumin-bound PTX in killing GC PDOs at both the transcriptome and cellular levels. Our results further showed that the different distributions of liposomal PTX and albumin-bound PTX in PDOs played an essential role in the distinct mechanisms through which they kill PDOs. Finally, we constructed patient-derived xenografts model in which we verified the above distinct therapeutic outcomes via an intratumoral administration route.

**Conclusions:**

This study demonstrates that GC PDOs are reliable tools for predicting nanoformulation efficacy.

**Graphical Abstract:**

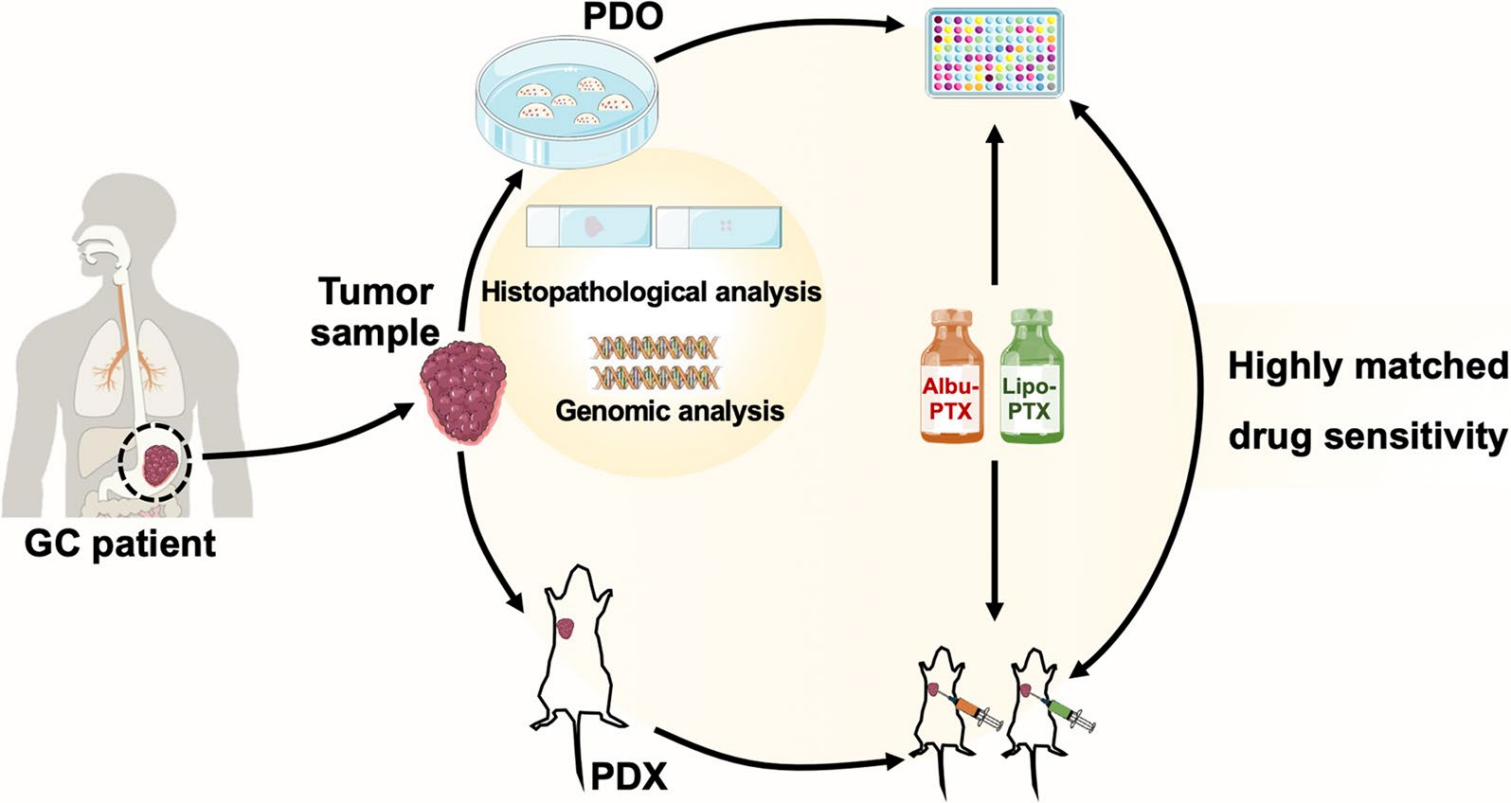

**Supplementary Information:**

The online version contains supplementary material available at 10.1186/s12951-022-01431-8.

## Introduction

Gastric cancer (GC) is the fifth most common cancer and the fourth most common cause of cancer death worldwide [1]. It is a highly heterogeneous disease with many different histological and molecular subtypes [2, 3]. Most GC patients have locally or distantly advanced disease at diagnosis [1], and chemotherapy is one of the primary methods used to treat the disease. Conventional chemotherapeutic agents have limited application in the clinic, mainly due to their undesirable systemic adverse effects [4]. For example, paclitaxel (PTX), one of the chemotherapeutic agents that is widely used in the treatment of GC patients, targets microtubules; it promotes microtubule polymerization and suppresses microtubule depolymerization, thereby ultimately arresting mitosis and triggering apoptosis [5]. In addition to PTX’s myelosuppression, cardiovascular toxicity and neurotoxicity, adverse effects that are shared with other chemotherapeutics, patients who receive traditional PTX formulations also suffer from a high incidence of hypersensitivity reactions [6]. This hypersensitivity has been shown to be highly correlated with the use of a nonaqueous vehicle containing Cremophor EL® (polyethoxylated castor oil) that is used to dissolve PTX and thereby facilitate its intravenous injection [7].

In recent years, the above dilemmas related to the use of chemotherapeutic agents have promoted research on the development of nanoformulations, including nanoemulsions, micelles, liposomes, dendrimers, et al. [8–11]. Excitingly, some of these have been approved for use in the clinic. Again taking PTX as an example, albumin-bound PTX (Albu-PTX) and liposomal PTX (Lipo-PTX) have been clinically used to treat patients with GC as well as individuals with other types of cancer [12, 13], and accumulating evidences have demonstrated their advantages in avoiding hypersensitivity reactions and reducing myelosuppression, cardiovascular toxicity and neurotoxicity [14–17]. However, the current clinical guidelines and expert consensus based on traditional PTX formulations may not be applicable to these newly emerging nanoformulations since they have unique in vivo features such as tumor uptake and tissue distribution [18, 19]. Moreover, the histological and molecular heterogeneity of GC also makes individual medication references and guidance necessary if we are to achieve further improved therapeutic outcomes.

Many researchers have devoted themselves to developing reliable models that can be used to predict the therapeutic responses of patients. For example, patient-derived xenografts (PDXs) have been used to evaluate the therapeutic efficacies of personalized tumor treatments in patients [20]. Recently, patient-derived organoids (PDOs), which are miniature, three-dimensional (3D), self-organized tissue culture models derived from primary patient tumor stem cells, have emerged as alternative tools [21–23]. PDOs closely recapitulate the genotypic, phenotypic, histological and malignant features of the corresponding primary tumors [24–26]. Compared with PDXs, PDOs have the advantages of rapid construction, high success rate, and high-throughput capacity [27, 28], all of which may help further accelerate the process of providing guidance for the rational and individualized use of anticancer therapeutic agents.

Keeping these points in mind, we envisioned that PDOs derived from GC patients could provide an ideal model for investigating the therapeutic responses to newly clinically used nanoformulations in the individual medical aspect. To this end, we constructed multiple GC PDOs lines from surgically resected tumor tissues and endoscopic biopsies, and the successful construction was systematically verified by histopathological and genomic profiles. We then comparatively evaluated the responses of these PDOs to two types of clinically used PTX nanoformulations, Albu-PTX and Lipo-PTX, and elucidated their underlying mechanisms of action. We also utilized the GC PDX model to reproduce distinct responses to Albu-PTX and Lipo-PTX, which further support the reliability of utilizing GC PDOs in evaluation of the efficacies of nanoformulations.

## Results

### Construction of GC PDOs

**Fig. 1:**
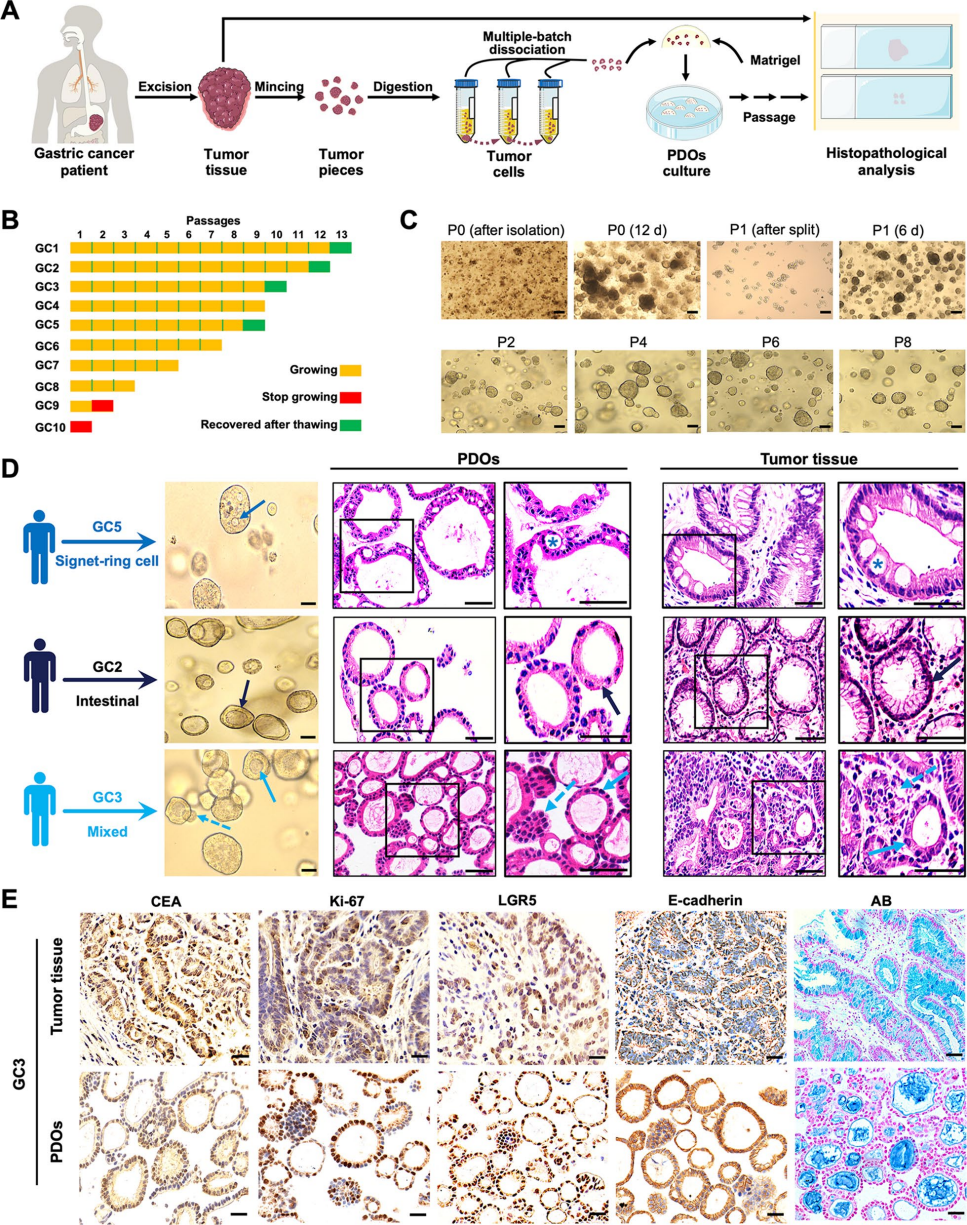
Construction and histopathological characterizations of GC PDOs. **A** Schematic overview of GC PDOs isolation, culture and histopathological analysis. **B** Number of passages and freeze-thaw status of GC PDOs derived from ten patients (numbered GC1 to GC10). Each block indicates one passage or a freeze-thaw cycle. For example, GC1 PDOs were passaged more than ten times and successfully thawed after freezing at passage 12, whereas GC9 PDOs stopped growing at passage 2. **C** Representative bright-field images of GC4 PDOs showing their morphological features during isolation, culture and passage. Scale bar, 100 μm. **D** Representative bright-field images and HE staining images of GC PDOs and the primary tumors from which they were derived. These tumors (GC5, GC2, and GC3) represent three histological subtypes. Scale bar, 50 μm. **E** Representative images showing IHC staining and AB staining of GC3 PDOs and the primary tumor. Scale bar, 30 μm.

To reliably evaluate the response of GC to nanoformulations *in vitro*, we began our study with PDOs construction. Primary tumor tissues were obtained from patients who were newly diagnosed with locally advanced gastric adenocarcinoma (Additional file 1: Table S1). According to methods described in previous reports [29, 30], tumor cells were liberated by enzymatic digestion of tumor pieces, plated in 3D Matrigel, and cultured in complete organoid medium (Fig. 1A). To avoid the microbial contamination, the GC samples were thoroughly washed prior to PDOs construction, and we added penicillin-streptomycin-amphotericin B to replace penicillin-streptomycin in the medium. To avoid the decreased cell viability caused by excessive digestion, we designed an improved enzymatic digestion process using multiple-batch dissociation instead of single-batch dissociation (Additional file 1: Fig. S1). During this process, we monitored the progress of digestion under a microscope. At intervals, dissociated cell clusters were harvested by centrifugation, and we repeated this process until significant cell dissociation from the tiny pieces was no longer observed. Typically, for 3–5 mm tumor pieces, 4–5 rounds of dissociation were required.

Using the improved approach, we constructed GC PDOs from tumor tissues derived from ten patients. Eight of these PDO lines (designated GC1-GC6, GC9-GC10) were sourced from resected tissues, and two (GC7-GC8) were obtained from endoscopic biopsies. As shown in Fig. 1B, nine PDO lines (GC1-GC9) were successfully constructed, and eight of them (GC1-GC8) were stably passaged. One PDO line (GC-9) stopped propagating after the first passage and another PDO line (GC-10) failed to form organoids after dissociation, which were attributed to microbial contamination originating from the surgically resected tissues. For the successfully passaged lines, 10–12 days were usually required for the primary culture to form mature PDOs (Fig. 1C). The lines could subsequently be passaged at split ratios of 1:3 to 1:2 approximately every 6–8 days thereafter. During long-term passaging, the morphology of the PDOs was well preserved (Fig. 1C). In addition to their passage ability, the organoids were well recovered after cryopreservation; this was verified in four PDO lines (GC1-GC3 and GC5) (Fig. 1B).

### Histopathological characterizations of GC PDOs

Given that GC has been demonstrated to display distinct histopathological subtypes [3], we chose PDOs derived from patients with three representative types of GC (GC2, intestinal gastric carcinoma; GC3, mixed gastric carcinoma; and GC5, gastric signet-ring cell carcinoma) and investigated the histopathological consistency of these PDOs with their primary tumors. Bright-field microscopic images of the three PDOs indeed showed distinct morphologies ranging from cystic structures with a thickened wall to compact structures with no lumen (Fig. 1D). Corresponding hematoxylin-eosin (HE) staining images further showed a strong concordance of the histopathological features of PDOs and those of their primary tumors. Specifically, GC5 PDOs established from signet-ring cell carcinoma reproduced the primary tumor’s ring-like appearance (indicated by an asterisk in the image) caused by displacement of the nucleus to one side of the cell by the large amount of mucin present in the tumor cell. For samples from GC2, both PDOs and the primary tissue exhibited glandular structures (indicated by the black arrow), a typical feature of intestinal gastric carcinoma. Further supporting the consistency in the morphology of the primary tumors and the PDOs derived from them, PDOs and primary tissue from GC 3 patients shared the features of mixed gastric carcinoma, showing glandular structures (indicated by the blue solid arrow) and solid structures (indicated by the blue dotted arrow). Above features of GC3 PDOs were also well preserved after cryopreservation and recovery (Additional file 1: Fig. S2).

Having demonstrated the recapitulation of intertumor heterogeneity by our PDOs, we next investigated whether the PDOs maintained the biological characteristics of the primary tumors from which they were derived. Accordingly, GC3 PDOs and the primary tumor tissue were subjected to immunohistochemical (IHC) staining for the GC marker carcinoembryonic antigen (CEA), the cell proliferation marker Ki-67, the gastrointestinal stem cell marker leucine-rich repeat-containing G protein-coupled receptor 5 (LGR5), and the epithelial cell marker E-cadherin. The staining results showed that the primary tumor’s markers were well maintained in PDOs (Fig. 1E). Alcian blue (AB) staining and Sirius red staining images showed similar patterns of distribution of extracellular components in PDOs and primary tumor (Fig. 1E and Additional file 1: Fig. S3). Furthermore, we also performed IHC staining on the recovered GC3 PDOs after cryopreservation, and found that they still maintained the biological characteristics of the primary tumor (Additional file 1: Fig. S4).

### Genomic characterizations of GC PDOs

**Fig. 2:**
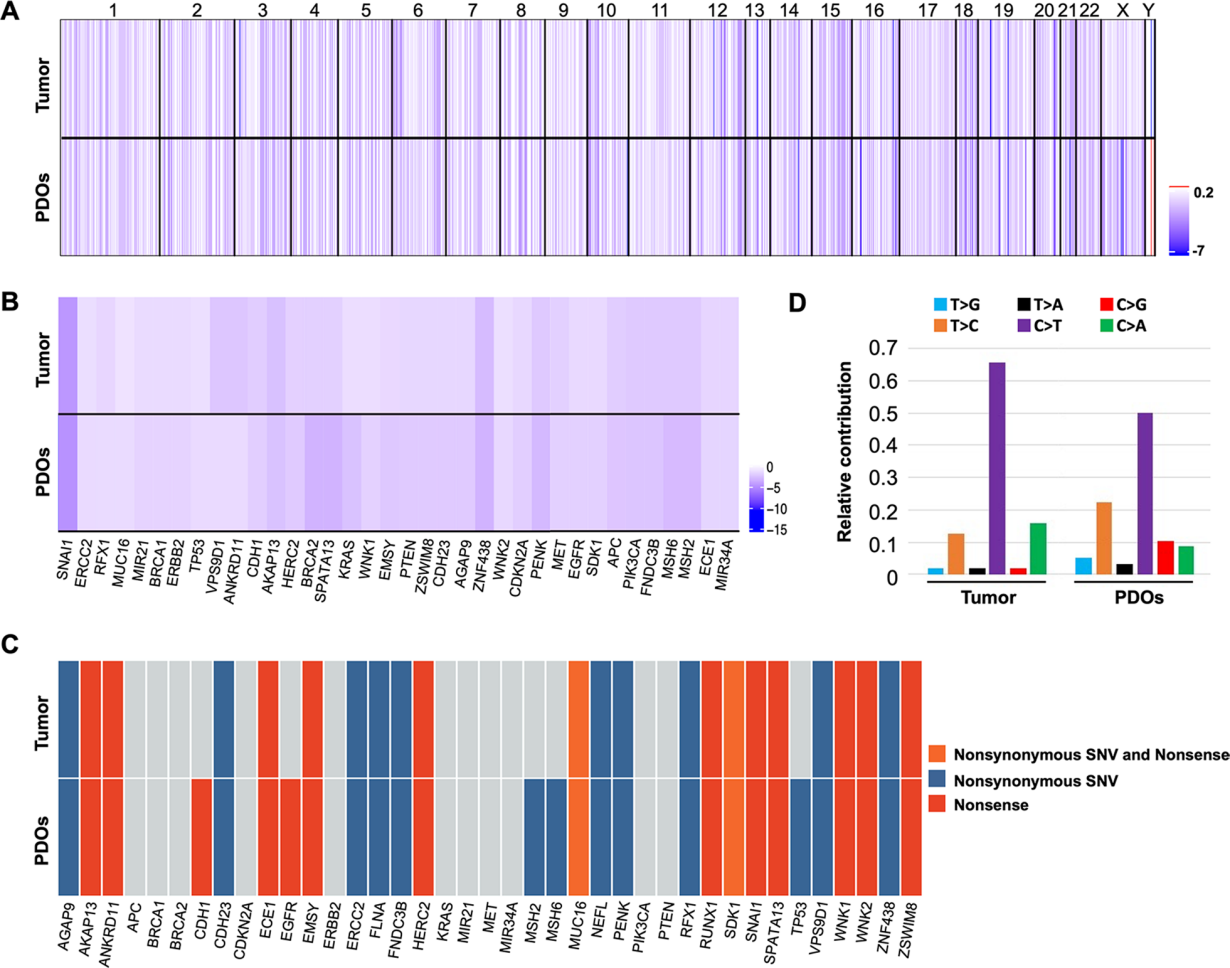
Genomic comparisons of GC PDOs with the primary tumor from patient GC3. **A** Heatmap showing CNVs in the primary tumor and the corresponding GC PDOs. The columns represent genomic positions from chromosomes 1 to 22, and the colors in the plot correspond to the estimated log_2_ copy ratios of the genomic regions. **B** CNVs heatmap of GC driver genes in the primary tumor and the corresponding GC PDOs. Gene copy numbers are transformed as log_2_ ratios per gene. **C** Heatmap showing gene mutation variations in the most frequently mutated GC genes. SNV: single-nucleotide variant. **D** Bar graphs showing the point mutation types found in the primary tumor and in the corresponding GC PDOs.

In addition to histopathological characterization of organoids, previous studies have also revealed that organoids can recapitulate the genomic profiles of their corresponding tumors [24, 25]. To validate this in our PDOs, we subjected GC3 PDOs and the primary tumor sample from which they were obtained to a series of genomic comparisons. Whole exome sequencing (WES) analysis revealed that GC3 PDOs largely recapitulated the primary tumor from which they were derived in terms of copy number variations (CNVs) (Fig. 2A). By searching the DriverDBv2 database, we further found that the CNVs of common driver genes of GC, such as *TP53*, *PIK3CA*, *CDH1*, and *KRAS*, exhibited a high degree of similarity in PDOs and primary tumor (Fig. 2B). Moreover, we conducted mutational spectrum analysis and found that PDOs retained most of the mutations in genes (GeneCards: the Human Gene Database) such as *ERCC2*, *SNAI1*, *HERC2*, and *RUNX1* that are observed in primary GC tumor (Fig. 2C). PDOs and the primary tumor also showed similarity with respect to the overall trend in the type of point mutations (Fig. 2D). Together, these results demonstrate that our established PDOs indeed recapitulate the genomic profiles of primary tumor.

### Efficacies of clinically used PTX nanoformulations on GC PDOs

**Fig. 3:**
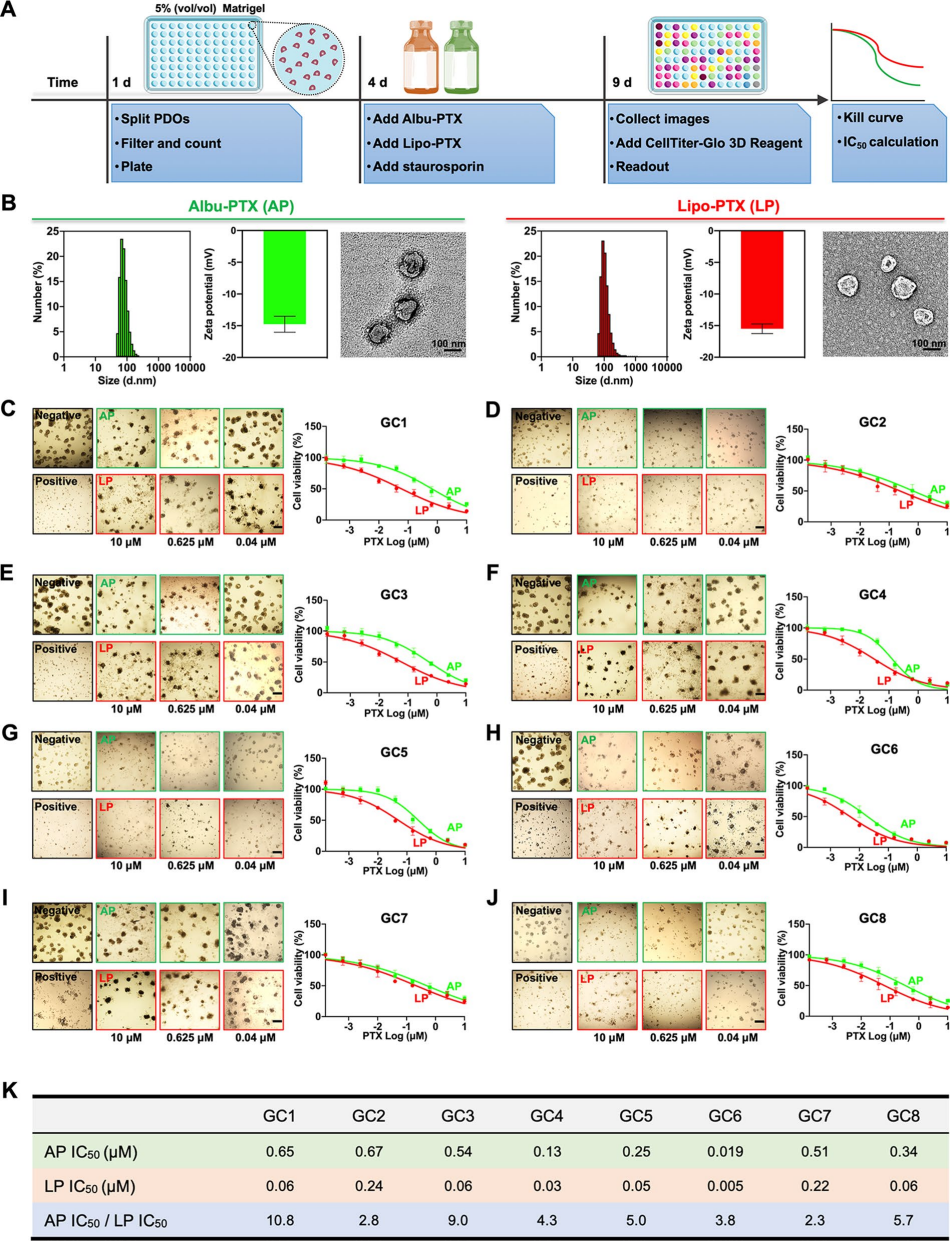
Response of GC PDOs to clinically used PTX nanoformulations. **A** Schematic overview of the drug sensitivity analysis protocol. **B** Size distributions, zeta potentials, and representative TEM images of Albu-PTX and Lipo-PTX. **C–J** Representative bright-field images and dose-response kill curves of various PDO lines treated with Albu-PTX or Lipo-PTX. PDOs were treated with PTX at concentrations ranging from 1.5 × 10^− 4^ µM to10 µM for 5 days. Scale bar, 200 μm. **K** IC_50_ values for various PDO lines. Data in (**B–J**) are presented as the means ± s.d (n = 3)

After successfully constructing GC PDOs, we comparatively investigated the efficacies of the clinically used PTX nanoformulations Albu-PTX and Lipo-PTX. To do this, we designed an improved protocol which was more suitable for working with nanoformulations. Briefly, single cells dissociated from the last passage were filtered through a 70-µm cell strainer; this step removed incompletely dissociated cell clusters and made it possible to dispense consistent numbers of cells into 96-well plates. In this experiment, we also utilized ultralow-attachment culture plates and decreased the Matrigel concentration in the complete organoid medium to 5% (vol/vol) to avoid restricting diffusion of the nanoformulations through the Matrigel. After confirming PDO formation three days later, Albu-PTX and Lipo-PTX were separately added to the culture medium at concentrations designed to produce the same PTX concentration gradients. Staurosporin (5 µM) was added to parallel wells as a positive control. After 5 days of treatment, bright-field images of PDOs were obtained, and intracellular adenosine triphosphate (ATP) levels were measured using CellTiter-Glo 3D Reagent to determine cell viability (Fig. 3A).

Before conducting the cell viability analysis, we characterized the two types of nanoformulations. Transmission electron microscopy (TEM) and dynamic light scanning (DLS) analysis showed that Albu-PTX and Lipo-PTX were very similar in size and had similar zeta potentials; for both formulations, these two parameters had narrow distributions that peak at approximately 100 nm and − 15 mV, respectively (Fig. 3B). After incubating the eight PDO lines with these two PTX nanoformulations, we found that the cluster size of PDOs decreased with increasing PTX concentration (Fig. 3C–J). Correspondingly, the kill curves for the eight PDO lines indicated that the cells displayed a concentration-dependent reduction in cell viability in response to exposure to Albu-PTX or Lipo-PTX.

Based on the kill curve data, we calculated the IC_50_ (half-maximal inhibitory concentration) of Albu-PTX or Lipo-PTX for the eight PDO lines; the results are summarized in Fig. 3K. We found that the eight PDO lines showed different responses to the two PTX nanoformulations. Specifically, the IC_50_ of Lipo-PTX for GC6 PDOs was 0.005 µM, while the corresponding IC_50_ for GC2 PDOs was 0.24 µM. Similarly, the IC_50_ of Albu-PTX in the PDO lines ranged from 0.019 µM to 0.67 µM, again emphasizing the intrapatient heterogeneity recapitulated by these PDO lines. On comparing the performances of the two nanoformulations in each PDO line, we observed that the IC_50_ values for Albu-PTX were always higher than those for Lipo-PTX. Quantitatively, the IC_50_ ratios of Albu-PTX/Lipo-PTX fluctuated from 2.3 to 10.8 in the eight PDO lines, indicating that Lipo-PTX outperformed Albu-PTX in killing the GC PDOs in our experiments.

### Mechanism of GC PDOs’ different responses to Albu-PTX and Lipo-PTX

**Fig. 4:**
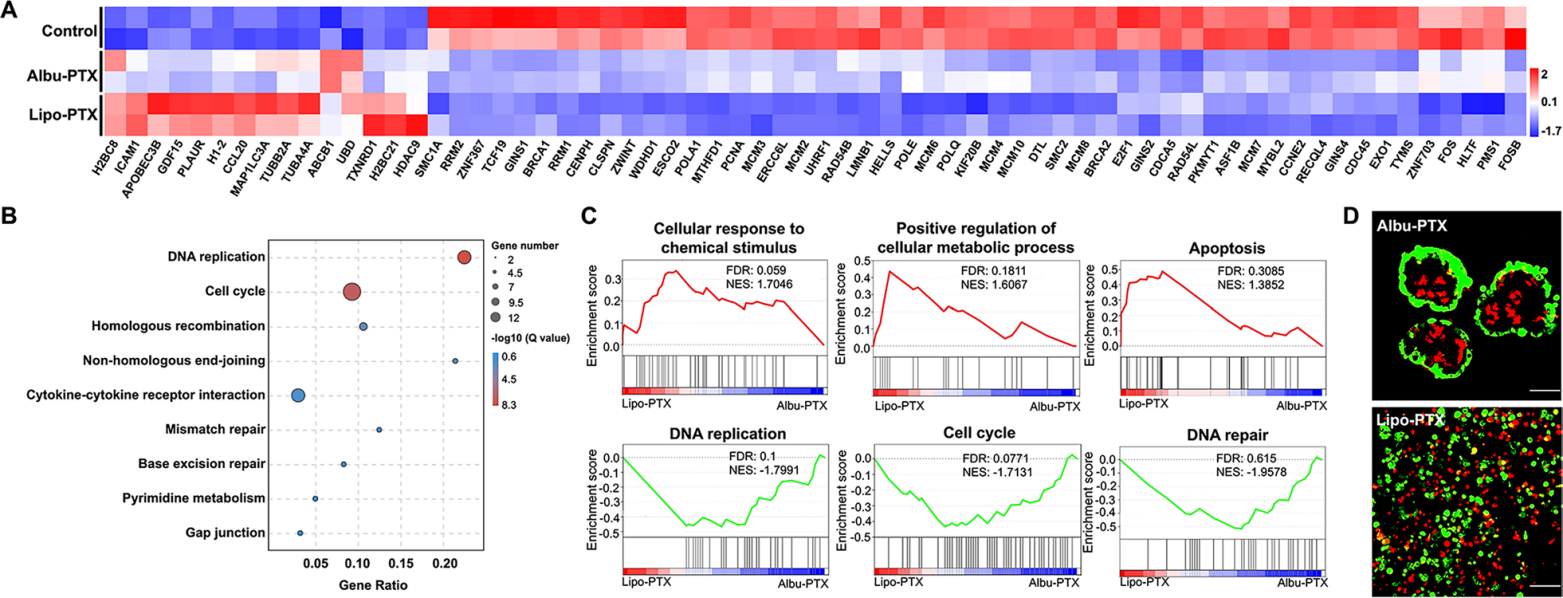
Distinct cytotoxic effects of PTX nanoformulations on GC1 PDOs at the transcriptome and cellular levels. **A** Transcriptome analysis of GC1 PDOs after treatment with Albu-PTX or Lipo-PTX. Differential gene cluster analyses are shown as heatmap. **B** KEGG pathway analysis of GC1 after treatment with Albu-PTX or Lipo-PTX. **C** GSEA of GC1 for gene sets that were changed in the Lipo-PTX treatment group versus the Albu-PTX treatment group. **D **Live-Dead staining analysis of GC1 PDOs after treatment with Albu-PTX or Lipo-PTX. Green: live cells; red: dead cells. Scale bar, 50 μm

The finding that Lipo-PTX and Albu-PTX have similar physicochemical properties but showed distinct killing efficacies raised our interest in investigating the mechanism underlying this difference. To this end, we selected GC1 PDOs, which showed the most distinct responses to the two PTX nanoformulations, for testing. Initially, we conducted transcriptome profiling analysis of GC1 PDOs after 48 h of treatment with Albu-PTX or Lipo-PTX at a PTX concentration of 0.04 µM. As shown in the heatmap of Fig. 4A, treatment with either Lipo-PTX or Albu-PTX increased the expression of several tubulin-related genes (including *TUBA4A* and *TUBB2A*) while decreasing the expression of many genes related to DNA replication and repair (including *MCM7*, *BRCA2*, and *POLE*). Kyoto encyclopedia of genes and genomes (KEGG) pathway analysis also revealed that exposure to these formulations affects pathways involved in DNA replication, the cell cycle and gap junctions (Fig. 4B). The above variations are consistent with PTX’s anticancer mechanism, which includes promotion of microtubule polymerization, suppression of microtubule depolymerization, and subsequent mitotic arrest.

Focusing on the comparison of the two PTX nanoformulations, we further found that the abovementioned genes in the heatmap were upregulated or downregulated to a greater extent in the Lipo-PTX group than in the Albu-PTX group. This prompted us to conduct additional gene set enrichment analysis (GSEA). Compared to the Albu-PTX group, the Lipo-PTX group particularly showed upregulation of sets of genes related to cellular responses to chemical stimulus, positive regulation of cellular metabolic process, and apoptosis and downregulation of sets of genes related to DNA replication, cell cycle, and DNA repair (Fig. 4C). Beyond the transcriptome aspect, we also performed Live-Dead staining analysis of GC1 PDOs. In the Albu-PTX group, the 3D structure of the whole PDO was well preserved, and only a few dead cells were detected inside the PDO lumen. In sharp contrast, treatment with Lipo-PTX induced substantial destruction of the 3D structure of the PDOs, and dead cells dominated the cell populations (Fig. 4D).

**Fig. 5:**
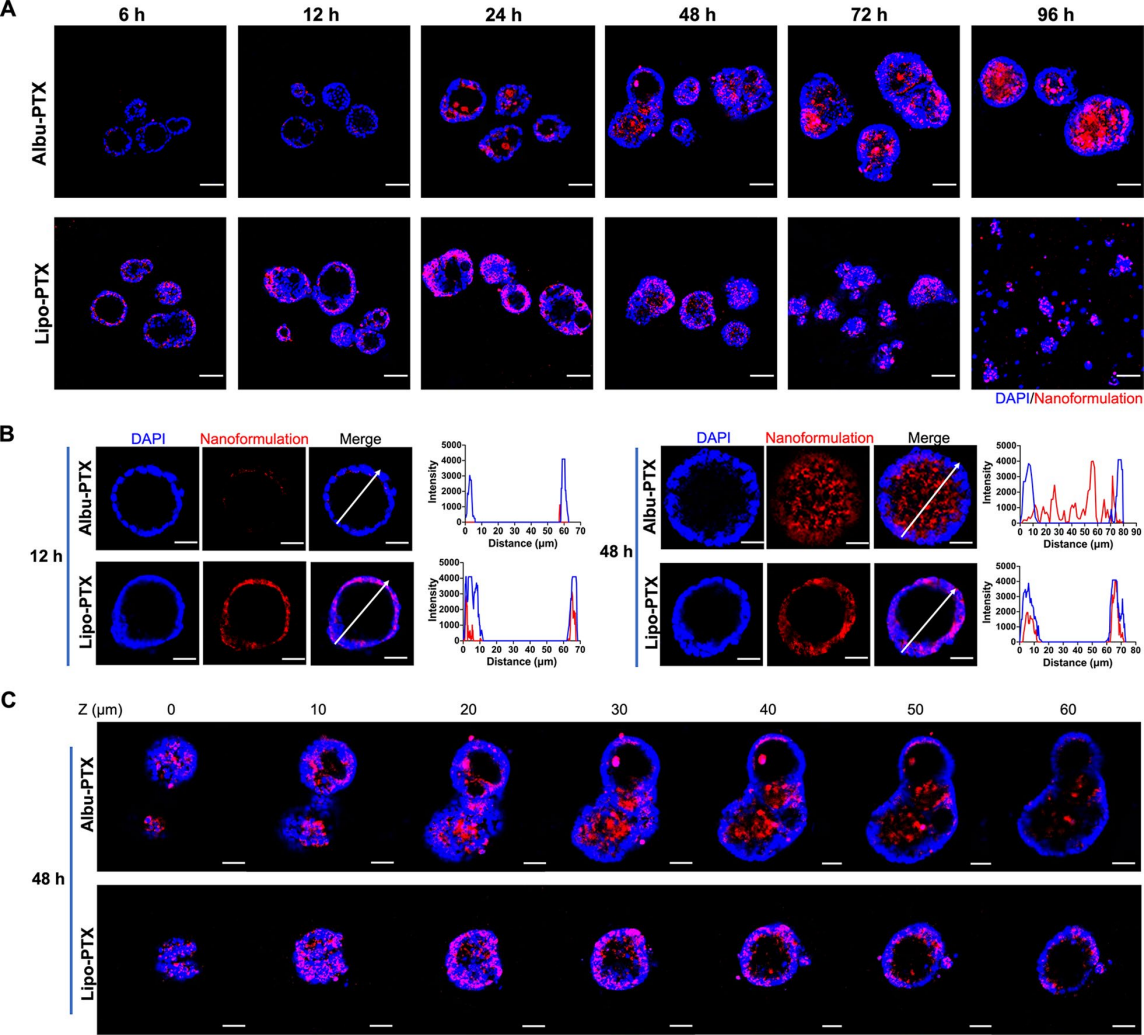
Distinct spatiotemporal distributions of PTX nanoformulations in GC1 PDOs. **A** The distributions of PTX nanoformulations in GC1 PDOs as revealed by CLSM at the indicated time points. Blue: DAPI; red: fluorescent-labeled Albu-PTX or Lipo-PTX. Scale bar, 50 μm. **B** The distributions of PTX nanoformulations in GC1 PDOs as revealed by fluorescence colocalization analysis. Blue: DAPI; red: fluorescent-labeled Albu-PTX or Lipo-PTX. Scale bar, 20 μm. **C** A series of Z-stack images showing the distributions of PTX nanoformulations in GC1 PDOs. Blue: DAPI; red: fluorescent-labeled Albu-PTX or Lipo-PTX. Scale bar, 30 μm

Based on the above Live-Dead pattern, we conjectured that the intra-PDO distributions of Albu-PTX and Lipo-PTX might correlate with their distinct PDO-killing performances. To pursue this, we labeled the nanoformulations with fluorescent dyes and monitored their distributions in GC1 PDOs by confocal laser scanning microscopy (CLSM). As shown in Fig. 5A, Albu-PTX began to accumulate in the PDO lumen at 24 h, and the PDOs continued to grow and were fully occupied by Albu-PTX until 96 h. In sharp contrast, image captured at 6 h revealed that Lipo-PTX distinctively appeared in the PDO cell layers rather than in the luminal cavity, and the amount of Lipo-PTX localized in the cell layers gradually increased over time until the PDOs lost their structural integrity. The distinct spatiotemporal distributions of Albu-PTX and Lipo-PTX were further confirmed by fluorescence colocalization analysis between nanoformulations and PDO cells (Fig. 5B) as well as a series of Z-stack images and corresponding 3D reconstruction data (Fig. 5C and Additional file 1: Fig. S5). Taken together, these results showed that the distinct distributions of Albu-PTX and Lipo-PTX in PDOs played essential roles in their differential performance in killing PDOs.

### Reproduction of differences in the therapeutic efficacies of PTX nanoformulations in the PDX model

**Fig. 6:**
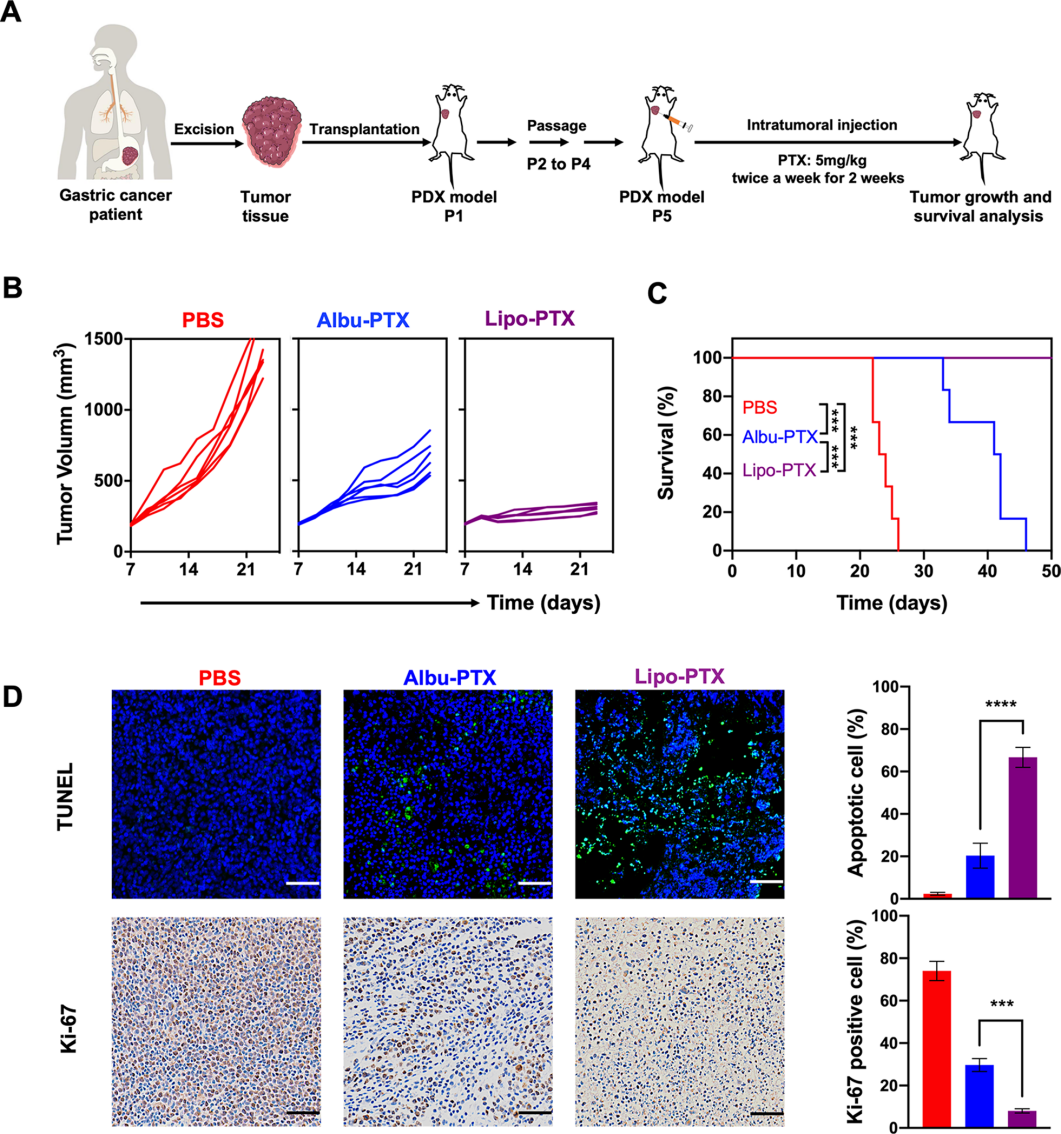
In vivo antitumor effects of intratumorally injected PTX nanoformulations in the GC1 PDX model. **A** Schematic illustration of GC1 PDX construction and the experimental design used in evaluation of the PDX response to intratumoral injection of PTX nanoformulations. **B** Individual growth kinetics of PDX tumors in different treatment groups (*n* = 6). **C** Survival curves of the mice in B. **D** TUNEL assay results and IHC analysis of Ki-67 expression in different treatment groups. Scale bar, 200 μm. Data in (**D**) are presented as the means ± s.d. (n = 3), and *P* values are determined by log-rank (Mantel-Cox) test (**C**) or one-way ANOVA with Tukey post-hoc test (D). ***P < 0.001 and ****P < 0.0001

Finally, we were in a position to determine whether the therapeutic effects of PTX nanoformulations observed in the GC PDO model could be reproduced in vivo. To this end, we again utilized GC1 primary tumor tissue to construct a PDX model for evaluation (Fig. 6A). Briefly, fresh tumor samples resected from GC1 patients were transplanted subcutaneously into NOD.Cg-Prkdc^scid^I12rg^tm1Vst^/Vst (NPG) mice. After engraftment for three passages, the tumor samples were transplanted into the armpits of NPG mice to establish the PDX model. When the tumor volumes reached approximately 180 mm^3^, the tumor-bearing NPG mice were divided randomly into three groups. The mice in the individual groups received four rounds of intratumoral injection of either phosphate-buffered saline (PBS), Albu-PTX (PTX dose: 5 mg/kg) or Lipo-PTX (PTX dose: 5 mg/kg). In this experiment, the use of clinical samples from the same source and direct exposure of the tumor cells to PTX nanoformulations ensured the rationality of comparing the results obtained in the PDO and PDX models.

Compared to the PBS group, in which tumors developed rapidly, treatment of the mice with either Albu-PTX or Lipo-PTX significantly inhibited tumor growth, and much more pronounced tumor inhibition was observed in the Lipo-PTX group than in the Albu-PTX group (Fig. 6B and Additional file 1: Fig. S6). Correspondingly, the survival rates for mice in the PBS, Albu-PTX and Lipo-PTX groups at 42 days were 0%, 16.7% and 100%, respectively (Fig. 6C). The above distinct therapeutic outcomes were further verified by histological analysis of additional mice at day 21 (Fig. 6D and Additional file 1: Fig. S7). In accordance with HE staining images, terminal-deoxynucleotidyl transferase-mediated dUTP nick end labeling (TUNEL) assays showed that only about 20% of the cells in Albu-PTX-treated PDX tissue were apoptotic, whereas this value increased to about 70% in the Lipo-PTX group. Reverse trends were found in Ki-67 (a cell proliferation marker) staining images. Collectively, these results support the idea that intratumoral injection of Lipo-PTX has superior therapeutic effects compared to Albu-PTX in the PDX model, which is consistent with our previous results in the PDO model.

## Discussion

In this study, we successfully established GC PDOs using a multiple-batch dissociation method to collect cells with improved viability. In addition to establishing PDOs from surgically resected tumor tissues, we also confirmed the feasibility of using endoscopic biopsies for GC PDO establishment, which has more significance in the clinic. As patients suspected of having GC always require upper endoscopic examination for diagnosis, endoscopic biopsies obtained in this scenario can provide clinical samples from diagnosed patients in a timely manner for subsequent PDO establishment and drug sensitivity screening. Moreover, specifically for middle-stage GC patients, early establishment of PDOs derived from endoscopic biopsies can provide useful drug sensitivity guidance for neoadjuvant chemotherapy prior to surgical resection as well as for adjuvant chemotherapy after surgical resection, whereas traditional PDO establishment from surgically obtained tumor tissue does not offer the opportunity to guide neoadjuvant chemotherapy. In addition, because GC patients with distant metastasis are not candidates for surgery, no surgical tumor tissue can be obtained from such patients. In this case, endoscopic biopsies are the most feasible source of tissue for PDO establishment, and subsequent drug sensitivity analysis may provide great benefit for improving the prognoses of these patients.

The GC PDOs we established closely recapitulated the features of their corresponding primary tumors. On the one hand, GC PDOs maintained the histopathological classification and cell markers of the tumors from which they were derived; on the other hand, the CNVs and the mutated genes associated with GC exhibited a high degree of similarity in PDOs and the corresponding primary tumors. With these PDOs in hand, we found that Lipo-PTX outperformed Albu-PTX in killing PDOs, and this was further verified at the transcriptome level. The different cytotoxicities of the two formulations were experimentally explained by the different distributions of Albu-PTX and Lipo-PTX in PDOs. We also used the GC PDX model to reproduce the therapeutic superiority of Lipo-PTX over Albu-PTX; the results again supported the idea that our PDOs is a reliable tool for predicting the efficacies of nanoformulations, which could be achieved within three weeks. It should be emphasized that, in the PDX model, the nanoformulations were administered via an intratumoral route; such direct exposure ensured the rationality of comparing the results obtained in the PDO and PDX models. Clinically, the intratumoral administration route may also be applicable during endoscopic manipulation. This approach can increase local drug concentrations in tumors and minimize systemic toxicity and has already been utilized as a palliative therapy to relieve symptoms and as an adjunct to systemic chemotherapy [31–33].

Although we herein demonstrated success in using our PDOs to predict the therapeutic outcomes of nanoformulations, efforts can be made to further improve GC PDOs in the future. For example, the PDOs obtained in our study and in other previous reports lack stromal cells such as immune cells and vascular endothelial cells [34, 35]. Therefore, they may not be good candidates for nanoformulations loaded with cisplatin or bevacizumab, both of which have strong effects on immunogenic cell death or anti-angiogenesis [36, 37]. One possible way to solve this problem would be to coculture PDOs with immune cells and vascular endothelial cells and thereby to closely recapitulate the tumor microenvironment [38–41]. Given that systemic intravenous administration is the main way in which chemotherapy for the treatment of malignant tumors is administered, PDOs can be further integrated with other modules to better recapitulate in vivo environments during the intravenous administration of nanoformulations. For example, two chambers that separately contain macrophages and PDOs can be connected in such a way that material from the two chambers is successively injected; this could work for predicting macrophage interception during blood circulation and intratumoral distribution after extravasation from blood vessels. Consideration of this point also raises the possibility that Albu-PTX may outperform Lipo-PTX when administered via intravenous injection. Therefore, it should again be emphasized that the focus of our study was the construction of GC PDOs and the feasibility of using such PDOs in the evaluation of nanoformulations per se rather than drawing a one-size-fits-all conclusion regarding the choice of Lipo-PTX over Albu-PTX. At minimum, it is clear that evaluation of the performance of nanoformulations using PDOs should become an integral step in experimental and clinical design.

## Methods

### Study approval

All human sample collections and experiments were reviewed and approved by the Chinese PLA General Hospital Medical Ethics Committee in accordance with the 1964 Declaration of Helsinki and 1982 International Ethical Guidelines for Human Biomedical Research (approval number: S2017-010-02), and informed consent was obtained from all of the participants. The mice experiment was approved by the Institutional Animal Care and Use Committee at the Institute of Process Engineering, Chinese Academy of Sciences (Approval Number: IPEAECA2021011).

### Construction, culture and passage of GC PDOs

Highly fibrotic, fatty, and severely necrotic tumor tissue should be avoided for the construction of PDOs. The clinically collected GC patient’s tumor samples should be conditioned in ice-cold PBS with 10 mM HEPES (03-025-1B, BI), 10 µM Rho kinase (ROCK) inhibitor compound Y-27632 (72302, Stemcell), and 100 U/mL penicillin-streptomycin-amphotericin B (P7630, Solarbio). The transportation time should be less than 24 h. When the tumor samples were transported to the laboratory, one part was fixed in 4% paraformaldehyde for histopathological and IHC analyses. Another part of tumor tissue, accompanied with corresponding para-carcinoma tissue, were quickly freezed in a liquid nitrogen tank for WES analysis. For construction of GC PDOs, GC patient’s tumor samples were washed with ice-cold PBS with 10 mM HEPES and 100 U/mL penicillin-streptomycin- amphotericin B at least five times. Then, we removed as much non-epithelial tissues as possible, and minced the tissues into small pieces and digested in 5 mL Dulbecco’s Modified Eagle’s Medium: Nutrient Mixture F-12 (DMEM/F12) (01-172-1ACS, BI) containing 1 mg/mL collagenase I (V900891-1, LABLEAD), II (V900892-1, LABLEAD), and IV (V900893-1, LABLEAD). During digestion, we mixed contents by shaking vigorously and by pipetting the mixture up and down using a P1000 pipette. In order to maximize cell viability, multiple-batch digestion was adopted. In detail, we monitored the digestion process under a microscope. When a certain amount of dissociated cell clusters was observed, we let the suspension stand for 2 min and collected supernatant for centrifugation (300* g*, 5 min, 4 ℃) to isolate cells. The remaining undissociated sedimentation was continued to be digested and repeated the above steps. Finally, collected cells were resuspended in precooling mixture of DMEM/F12 and Matrigel (356231, Corning) at the ratio of 1:1, and seeded in a pre-heated 24-well flat bottom cell culture plate (35241, Corning) in drops of 50 µL each. After the drops have solidified, 750 µL IntestiCult Organoid Growth Medium (06010, Stemcell) was added to each well. Organoids were cultured in incubator at 37 ℃, 5% CO_2_. Medium was refreshed every 2–3 days.

GC PDOs were passaged with a split ratio of 1:3 to 1:2 approximately every 6–8 days. The organoids were mechanically pipetted out of Matrigel using cold DMEM/F12, and the organoids suspension was centrifuged at 300* g* for 5 min at 4 ℃. Then, the organoids were dissociated by TrypLE (12604-013, Gibco), and pipetted up and down to aid disruption of the organoids. Dissociation ended when cell clusters (consisting of 2–10 cells) can be observed under microscope. Cell clusters were collected by centrifugation (300* g*, 5 min, 4 ℃), re-suspended in precooling mixture of DMEM/F12 and Matrigel and then seeded as described above.

### Cryopreservation and recovery of GC PDOs

The medium was removed 2–3 days after splitting. The organoids were mechanically pipetted out of Matrigel using cold DMEM/F12, and the organoids suspension was centrifuged at 300* g* for 5 min at 4 ℃. Then, we aspirated the supernatant and added the cell-freezing medium (07930, Stemcell) to resuspend the organoids, and transferred the suspension to the cryogenic vial. The cryogenic vial was placed into a freezing container which was stored at − 80 ℃ for 24 h. Finally, the freezing container was transferred to the liquid nitrogen tank.

Before thawing of cryopreserved organoids, we prepared a 15-mL conical tub with 10 mL of DMEM/F12 at room temperature. Then, we removed the cryogenic vial from the liquid nitrogen tank, and incubated the cryogenic vial in a water bath at 37 ℃. The cryogenic vial was removed from the water bath when there was a small clump of ice in it. Finally, we transferred the suspension from the cryogenic vial to the previously prepared 15-mL conical tub. The organoids suspension was centrifuged at 300* g* for 5 min at 4 ℃, and re-suspended in precooling mixture of DMEM/F12 and Matrigel and then seeded as described above.

### HE and IHC staining of GC PDOs and primary tumor tissues

Tumor samples and PDOs pellets were fixed in 4% paraformaldegyde at 4 ℃ for 24 h and 30 min, respectively, and then followed by dehydration, paraffin embedding, sectioning and standard HE staining. IHC were performed for LGR5 (1:100, Affinity DF2816), E-cadherin (1:2000, proteintech 20874-1-AP), Ki-67 (1:10000, proteintech 27309-1-AP), and CEA (1:200, Bioss bs-0060R). Briefly, paraffin sections were deparaffinized in xylene and rehydrated through a graded ethanol series. After heat-mediated antigen retrieval with a sodium citrate buffer (10 mM, PH 6.0), the sections were blocked at room temperature for 30 min. The primary antibodies were diluted in 3% bull serum albumin (BSA), and staining was performed overnight at 4 ℃. Sections were then incubated with secondary antibodies at room temperature for 60 min. HE and IHC images were viewed and captured using an automatic multispectral imaging system (PerkinElmer Vectra II, USA).

### WES analysis

Genomic DNA was fragmented using NEBNext dsDNA Fragmentase (NEB, Ipswich, MA, USA) following by DNA ends repairing. End-repaired DNA fragments were dA-tailed and ligated with the NEBNext adaptor (NEB, Ipswich, MA, USA). Biotinylated RNA library baits and magnetic beads were mixed with the barcoded library for targeted regions selection using the SureSelect Human All Exon V6 Kit (AgilentTechnologies, Palo Alto, Calif.). The captured sequences were further amplified for 150 bp paired-end sequencing in Illumina X-ten system (Gene Denovo Biotechnology Co. China). To identify somatic SNV, the Burrows-Wheeler Aligner (BWA) was used to align the clean reads from each sample against the human reference genome (GRCh38). Somatic CNV was identified using VarScan 2 with the following parameter: phred base quality ≥ 20, minimum coverage ≥ 20. Mutational signatures were deciphered by using a non-negative matrix factorization method.

### RNA-sequencing analysis

Total RNA was extracted using Trizol method. After total RNA was extracted, eukaryotic mRNA was enriched by Oligo (dT) beads, while prokaryotic mRNA was enriched by removing rRNA by Ribo-Zero™ Magnetic Kit (Epicentre, Madison, WI, USA). Then the enriched mRNA was fragmented into short fragments using fragmentation buffer and reverse transcripted into cDNA with random primers. Second-strand cDNA were synthesized by DNA polymerase I, RNase H, dNTP and buffer. Then the cDNA fragments were purified with QiaQuick PCR extraction kit (Qiagen, Venlo, The Netherlands), end repaired, poly (A) added. The transcriptome sequencing was performed using Illumina HiSeq2500 (Gene Denovo Biotechnology Co. China). RNA expression levels were determined using the fragments per kilobase of transcript per million mapped reads method. The fold-change method was used to identify RNAs that were differentially expressed after castration using the R package DEGseq (R-3.6.2). Genes with a log_2_ fold change > 2 and an adjusted *P* < 0.05 were deemed to be significantly differentially expressed.

### Characterization of PTX nanoformulations

Images of PTX nanoformulations were obtained using a JEM-1400 TEM (JEOL, Japan) with an accelerating voltage of 120 KV. The size distribution and zeta potential of PTX nanoformulations were analyzed by DLS system (Malvern ZEN 3600 Zeta sizer, UK).

### Drug testing on GC PDOs

Single cells dissociated by TrypLE from the last passage PDOs were filtered through a 70-µm cell strainer. After cell counting, we resuspended these cells in IntestiCult Organoid Growth Medium containing 5% (vol/vol) Matrigel and then dispensed them in ultra-low attachment 96-well plate (3474, Corning) at 2000 cells per well. Organoids grew in 96-well plate for 3 days and then were treated with PTX nanoformulations (Albu-PTX and Lipo-PTX) at concentrations of 10, 2.5, 0.625, 0.16, 0.04, 0.01, 0.0025, 0.0006, and 0.00015 µM. The two PTX nanoformulations were commercial drugs used in the clinic, and we referred to the drug instructions to determine the content of PTX in nanoformulations (1000 mg Albu-PTX contains 100 mg of PTX, 1200 mg Lipo-PTX contains 30 mg of PTX). 5 µM staurosporin (HY-15141, MCE) treated wells were set as a positive control, and PBS treated wells were set as a negative control. Organoids cell viability was evaluated after 5 days drugs treatment. In detail, after adding CellTiter-Glo 3D Reagent (G9683, Promega) to the drug-screening plates, we performed the readout by measuring the luminescence intensity of each well. Then, we calculated the average luminescence value of the negative and positive control wells. Furthermore, we set the positive control to 0% viability and the negative control as 100%, and calculated the viability for each well according to the following formula:

Well viability =$$\frac{{{\text{Well}}\,{\text{value}} - {\text{Average positive control}}}}{{{\text{Average negative control}} - {\text{Average positive control}}}}*100\%$$  

Finally, we transferred the well viability to the GraphPad Prism 9.0.0 software, and chose the option “log (inhibitor) vs normalized response-variable slope” to create a dose-response kill curve.

### Live-Dead staining analysis

Live-Dead staining (C2015S, Beyotime) was carried out for GC PDOs after treatment with Albu-PTX or Lipo-PTX at a PTX concentration of 0.04 µM. Organoids were incubated for 30 min in a solution of calcein-AM and PI, according to the manufacturer’s instructions. Live cells (green fluorescence, 488 nm) and dead cells (red fluorescence, 550 nm) were simultaneously detected under a CLSM (Nikon A1R, Japan).

### Intra-PDO distributions of PTX nanoformulations

We labeled the PTX nanoformulations with fluorescent dyes and monitored their distributions in PDOs by CLSM. In detail, Albu-PTX was incubated with Cy5-SE (GC35771, GLPBIO), and Lipo-PTX was labeled with DiD (D22031, LABLEAD) at 37 ℃ for 2 h. After complete removal of free dye, the fluorescence intensities of Cy5-labeled Albu-PTX and DiD-labeled Lipo-PTX were detected to confirm the comparative fluorescence signal intensity (Excitation: 638 nm, Emission: 670 nm). Then the two kinds of fluorescent-labeled nanoformulations were separately added to the culture medium at a PTX concentration of 0.04 µM. Before monitoring the distributions of PTX nanoformulations in PDOs by a CLSM at the indicated time point (6 h, 12 h, 24 h, 48 h, 72 h, and 96 h after adding the PTX nanoformulations), the PDO cell nucleus was labeled with 4′,6-diamidino-2-phenylindole (DAPI) for 5 min. The same image acquisition parameters (include pinhole size, detector gain, amplifier offset/gain, scan speed/average, zoom, and laser intensity) were used when photographing different samples with CLSM (Nikon A1R, Japan). The fluorescence colocalization analysis between nanoformulations and PDO cells as well as a series of Z-stack images and corresponding 3D reconstruction data were acquired by the software of CLSM.

### Drug testing on GC PDX

Fresh GC patient’s tumor samples were conditioned in ice-cold PBS with 10 mM HEPES, 10 µM ROCK inhibitor compound Y-27632, and 100 U/mL penicillin-streptomycin-amphotericin B. When the tumor samples were transported to the laboratory, they were cut into 25–50 mm^3^ pieces and then transplanted subcutaneously into NPG mice (female, 6–8 weeks, Beijing Vital River Laboratory, China) to establish the PDX model. After the engraftment for three passages, the tumor samples were transplanted into the armpits of NPG mice for drug testing experiment. When the tumor volumes reached about 180 mm^3^, tumor-bearing NPG mice were allocated randomly to three groups and received four round intratumoral injection of PBS, Albu-PTX or Lipo-PTX. Albu-PTX was diluted in 0.9% Sodium Chloride Injection and Lipo-PTX was diluted in 5% Glucose Injection. For each injection, the PTX dose was 5 mg/kg, and the total volume was 100 µL. The tumor burden was monitored with a digital caliper every 2 days. The tumor volume was calculated using the following formula: Volume (mm^3^) = length × width^2^ / 2. Mice were considered dead when the tumor volume reached 1500 mm^3^.

### Statistical analyses

Statistical analyses were performed using GraphPad Prism 9.0.0 software. A two-tailed Student’s *t* test was used to compare two groups, one-way ANOVA with Tukey post-hoc test was used for the multi-group comparison, and log-rank test was used for the survival comparison. *P* < 0.05 was considered significant; significant values were indicated as **P < 0.01, ***P < 0.001, and ****P < 0.0001.

## Supplementary Information

**Additional file 1: Figure S1.** An improved enzymatic digestion process using multiple-batch dissociation instead of single-batch dissociation. **Figure S2.** Representative bright-field image and HE staining image of GC3 PDOs recovered after cryopreservation. The blue solid arrow indicates the glandular structure and the blue dotted arrow indicates the solid structure. Scale bar, 50 μm. **Figure S3.** Representative Sirius red staining images of GC3 PDOs and primary tumor. Scale bar, 50 μm. **Figure S4.** Representative images showing IHC staining of GC3 PDOs recovered after cryopreservation. Scale bar, 50 μm. **Figure S5.** Representative 3D reconstruction images showing the distributions of PTX nanoformulations in GC1 PDOs. Blue: DAPI; red: fluorescent-labeled Albu-PTX or Lipo-PTX. Scale bar, 30 μm. **Figure S6.** Growth kinetics of PDX tumors in different groups (n=6). Data are presented as the means ± s.d., and *P* values are determined by two-way ANOVA with Bonferroni post-hoc test. **P < 0.01 and ****P < 0.0001. **Figure S7.** Representative HE staining images of PDX tumors in different treatment groups. Scale bar, 200 μm. **Table S1.** Summary of clinical data of patients whose tumor tissues were used for PDOs construction.

## Data Availability

The datasets used and analyzed during the current study are available from the corresponding author on reasonable request.

## Abbreviations

GC: Gastric cancer
PTX: Paclitaxel
Albu-PTX: Albumin-bound PTX
Lipo-PTX: Liposomal PTX
PDXs: Patient-derived xenografts
PDOs: Patient-derived organoids
3D: Three-dimensional
HE: Hematoxylin-eosin
IHC: Immunohistochemical
CEA: Carcinoembryonic antigen
LGR5: Leucine-rich repeat-containing G protein-coupled receptor 5
AB: Alcian blue
WES: Whole exome sequencing
CNVs: Copy number variations
TEM: Transmission electron microscopy
DLS: Dynamic light scanning
IC_50_: Half-maximal inhibitory concentration
KEGG: Kyoto encyclopedia of genes and genomes
GSEA: Gene set enrichment analysis
CLSM: Confocal laser scanning microscopy
PBS: Phosphate-buffered saline
TUNEL: Terminal-deoxynucleotidyl transferase-mediated dUTP nick end labeling
