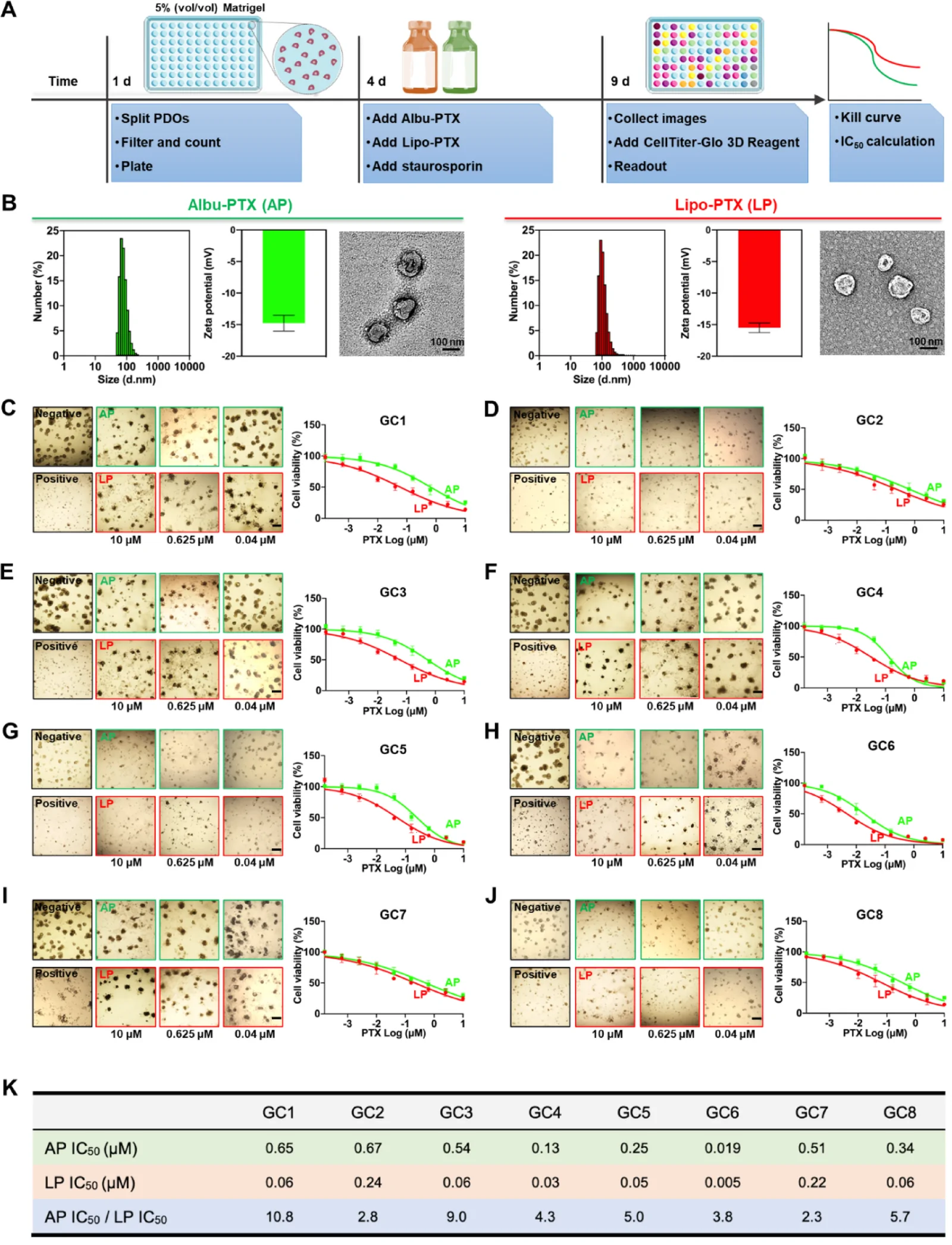# Correction: Construction of gastric cancer patient-derived organoids and their utilization in a comparative study of clinically used paclitaxel nanoformulations

**DOI:** 10.1186/s12951-026-05104-8

**Published:** 2026-10-07

**Authors:** Jiale Zou, Shuang Wang, Ningli Chai, Hua Yue, Peng Ye, Peilin Guo, Feng Li, Bo Wei, Guanghui Ma, Wei Wei, Enqiang Linghu

**Affiliations:** 1 Department of Gastroenterology and Hepatology, The First Medical Centre, Chinese PLA General Hospital, Beijing, 100853, People’s Republic Of China; 2 State Key Laboratory of Biochemical Engineering, Institute of Process Engineering, Chinese Academy of Sciences, Beijing, 100190, People’s Republic Of China; 3 School of Chemical Engineering, University of Chinese Academy of Sciences, Beijing, 100049 People’s Republic Of China; 4 Department of General Surgery, The First Medical Centre, Chinese PLA General Hospital, Beijing, 100853 People’s Republic Of China

**Correction: Journal of Nanobiotechnology (2022) 20:233**

**https://doi.org/10.1186/s12951-022-01431-8**

Figure 3 comprised 64 individual images and underwent multiple rounds of figure assembly and layout revision. During one of these steps, the intended image for the AP 0.625 µM condition in Fig. 3H appears to have been inadvertently misplaced. After the discrepancy was detected, we re-examined the original, uncropped imaging files for all eight conditions shown in Fig. 3H and located and verified the correct image for the AP 0.625 µM condition. The error was limited to image placement during figure assembly and did not affect the underlying experimental data, quantitative analyses, results, or conclusions of the article.

For completeness and transparency, the old incorrect and correct versions of Fig. 3 are displayed below. The original article has been corrected.

Incorrect Fig. 3.

**Figure:**
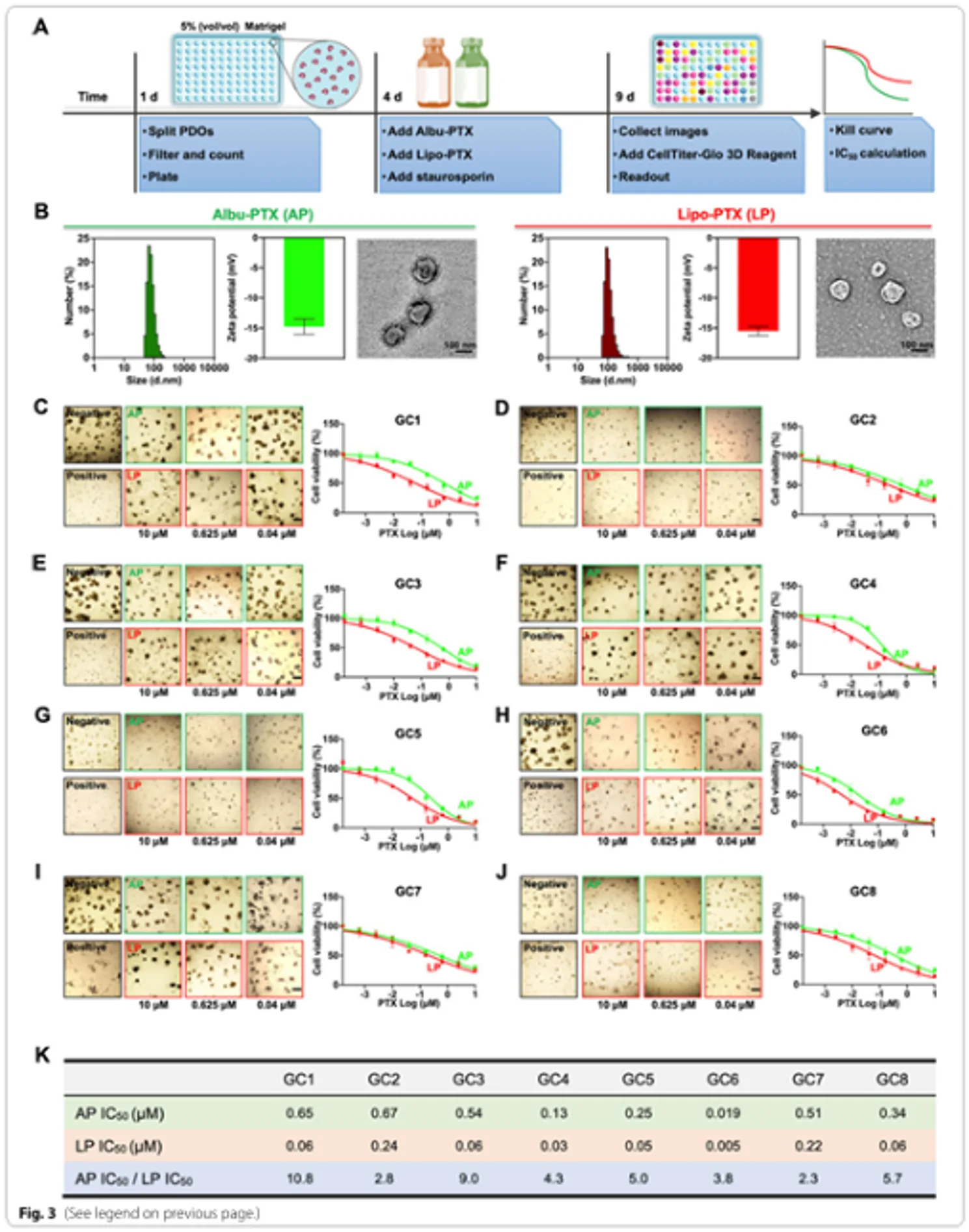

Correct Fig. 3.

**Figure:**