# Supplementary material for: Construction of gastric cancer patient-derived organoids and their utilization in a comparative study of clinically used paclitaxel nanoformulations
Source: J Nanobiotechnology. 2022 May 18;20:233. doi: 10.1186/s12951-022-01431-8 (PMC9118843; doi:10.1186/s12951-022-01431-8)
Supplement: Supplementary file 1 — Additional file 1: Figure S1. An improved enzymatic digestion process using multiple-batch dissociation instead of single-batch dissociation. Figure S2. Representative bright-field image and HE staining image of GC3 PDOs recovered after cryopreservation. The blue solid arrow indicates the glandular structure and the blue dotted arrow indicates the solid structure. Scale bar, 50 μm. Figure S3. Representative Sirius red staining images of GC3 PDOs and primary tumor. Scale bar, 50 μm. Figure S4. Representative images showing IHC staining of GC3 PDOs recovered after cryopreservation. Scale bar, 50 μm. Figure S5. Representative 3D reconstruction images showing the distributions of PTX nanoformulations in GC1 PDOs. Blue: DAPI; red: fluorescent-labeled Albu-PTX or Lipo-PTX. Scale bar, 30 μm. Figure S6. Growth kinetics of PDX tumors in different groups (n=6). Data are presented as the means ± s.d., and P values are determined by two-way ANOVA with Bonferroni post-hoc test. **P < 0.01 and ****P < 0.0001. Figure S7. Representative HE staining images of PDX tumors in different treatment groups. Scale bar, 200 μm. Table S1. Summary of clinical data of patients whose tumor tissues were used for PDOs construction. [file 12951_2022_1431_MOESM1_ESM.docx]

**Construction of gastric cancer patient-derived organoids and their utilization in a** **comparative study of clinically used paclitaxel nanoformulations**

Jiale Zou^1,2†^, Shuang Wang^2†^, Ningli Chai^1†^, Hua Yue^2^, Peng Ye^2^, Peilin Guo^2,3^, Feng Li^2,3^, Bo Wei^4^, Guanghui Ma^2,3*^, Wei Wei^2,3*^, Enqiang Linghu^1*^

^1^Department of Gastroenterology and Hepatology, The First Medical Centre, Chinese PLA General Hospital, Beijing 100853, P. R. China.

^2^State Key Laboratory of Biochemical Engineering, Institute of Process Engineering, Chinese Academy of Sciences, Beijing 100190, P. R. China.

^3^School of Chemical Engineering, University of Chinese Academy of Sciences, Beijing 100049, P. R. China.

^4^Department of General Surgery, The First Medical Centre, Chinese PLA General Hospital, Beijing 100853, P. R. China.

^†^These authors contributed equally to this work.

*Corresponding author email: ghma@ipe.ac.cn; weiwei@ipe.ac.cn; linghuenqiang@vip.sina.com

S1


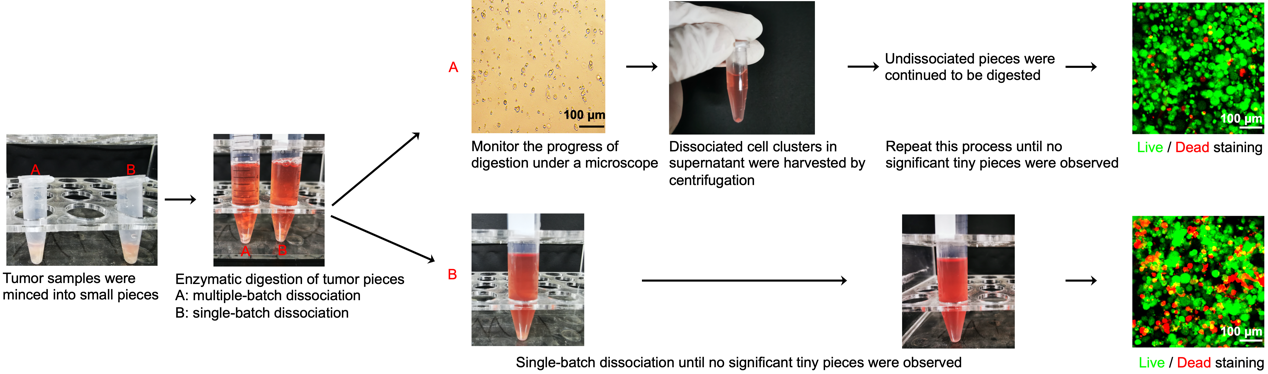


**Figure S1** An improved enzymatic digestion process using multiple-batch dissociation instead of single-batch dissociation.

S2


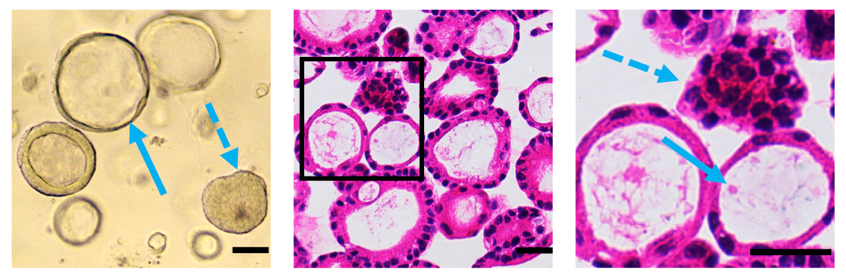


**Figure S2** Representative bright-field image and HE staining image of GC3 PDOs recovered after cryopreservation. The blue solid arrow indicates the glandular structure and the blue dotted arrow indicates the solid structure. Scale bar, 50 μm.

S3


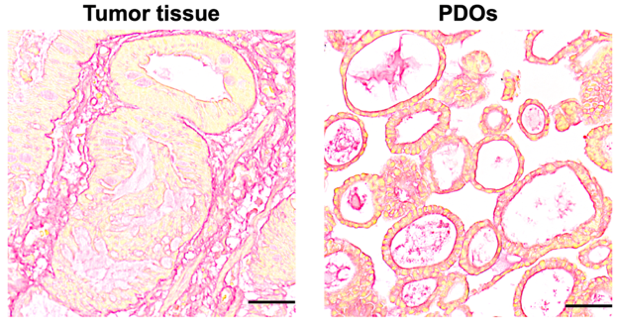


**Figure S3** Representative Sirius red staining images of GC3 PDOs and primary tumor. Scale bar, 50 μm.

S4


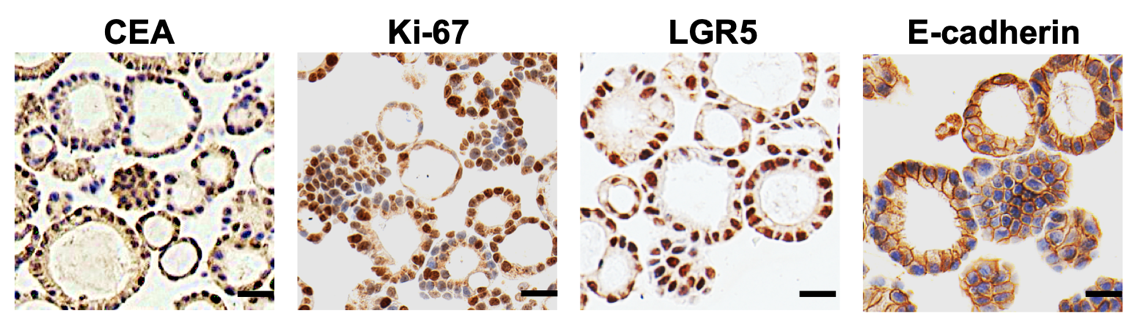


**Figure S4** Representative images showing IHC staining of GC3 PDOs recovered after cryopreservation. Scale bar, 50 μm.

S5


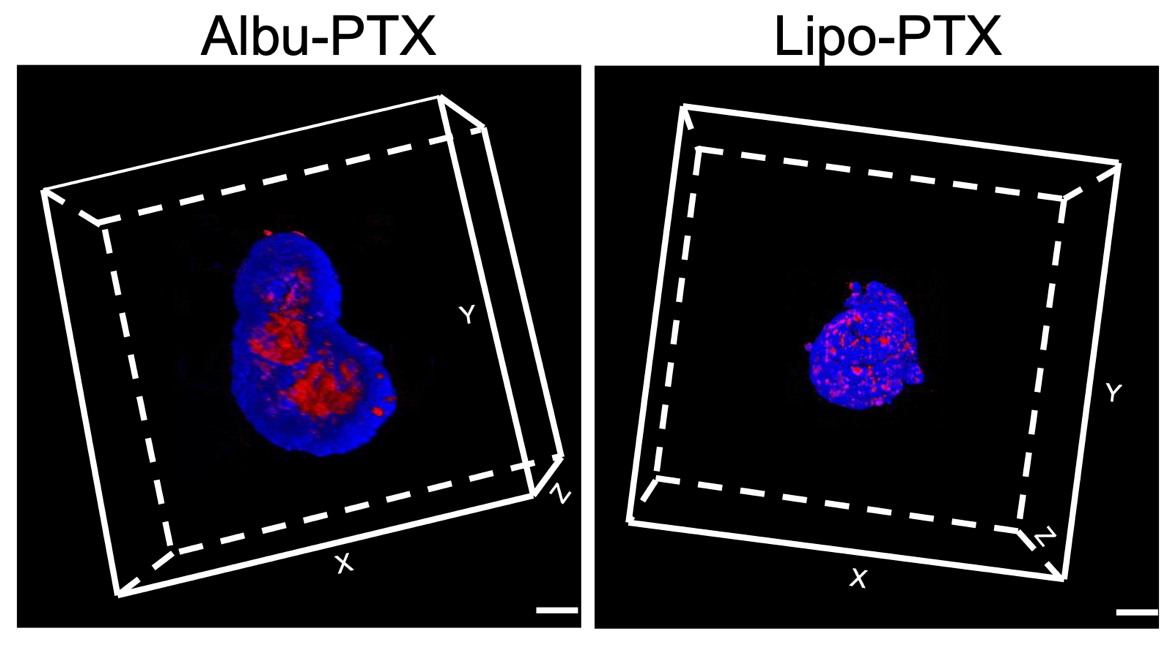


**Figure S5** Representative 3D reconstruction images showing the distributions of PTX nanoformulations in GC1 PDOs. Blue: DAPI; red: fluorescent-labeled Albu-PTX or Lipo-PTX. Scale bar, 30 μm.

S6


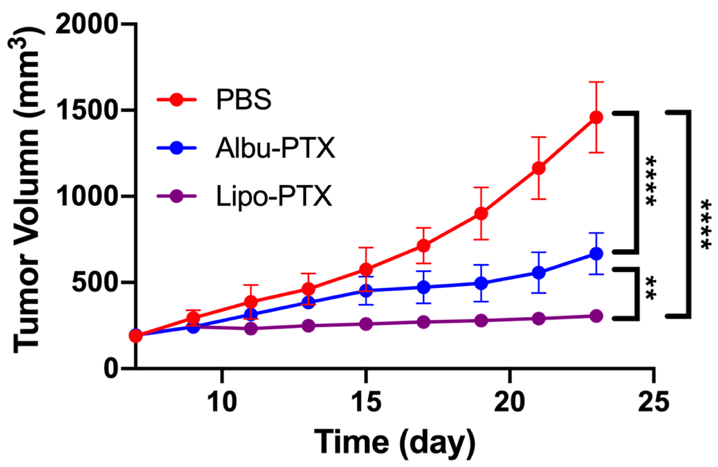


**Figure S6** Growth kinetics of PDX tumors in different groups (*n*=6). Data are presented as the means ± s.d., and *P* values are determined by two-way ANOVA with Bonferroni post-hoc test. ***P* < 0.01 and *****P* < 0.0001.

S7


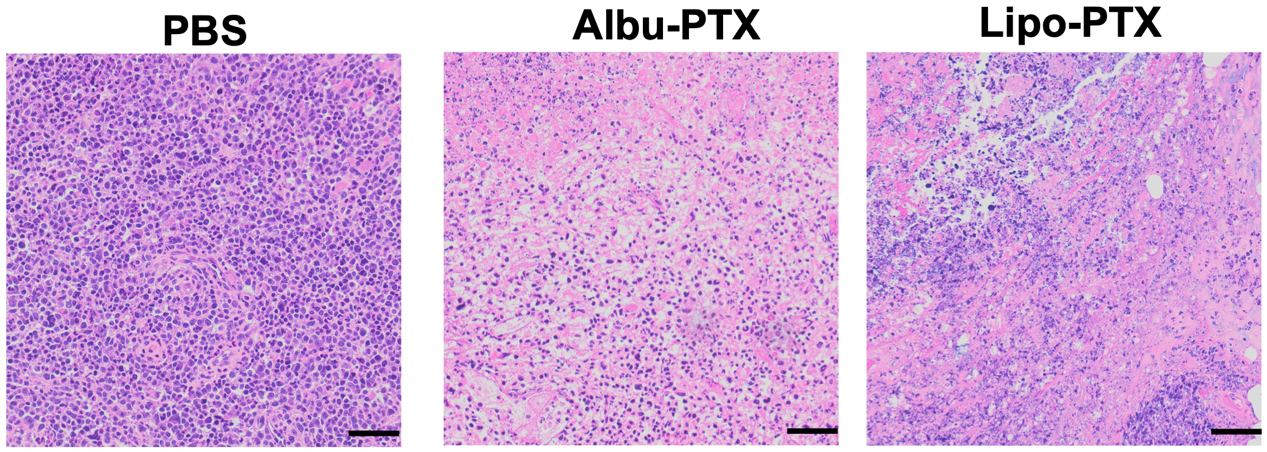


**Figure S7** Representative HE staining images of PDX tumors in different treatment groups. Scale bar, 200 μm.

**Table S1 Summary of clinical data of patients whose tumor tissues were used for PDOs construction**

| Patients | Sex | Age | Pathological classification | TNM Stage |
| --- | --- | --- | --- | --- |
| GC1 | Male | 73 | Poorly differentiated adenocarcinoma | T3N3aM0 |
| GC2 | Female | 63 | Moderately and poorly differentiated adenocarcinoma | T3N0M0 |
| GC3 | Female | 53 | Moderately differentiated adenocarcinoma | T4bN0M0 |
| GC4 | Male | 73 | Moderately and poorly differentiated adenocarcinoma | T3N3aM0 |
| GC5 | Female | 49 | Signet-ring cell carcinoma | T4aN2M0 |
| GC6 | Female | 70 | Moderately and poorly differentiated adenocarcinoma | T4bN3aM0 |
| GC7 | Female | 60 | Poorly differentiated adenocarcinoma | T4bN3aM0 |
| GC8 | Male | 48 | Poorly differentiated adenocarcinoma | T4bN2M0 |
| GC9 | Male | 63 | Moderately and poorly differentiated adenocarcinoma | T3N0M0 |
| GC10 | Male | 58 | Poorly differentiated adenocarcinoma | T4aN2M0 |
